# Potential Effectiveness of Chinese Patent Medicine *Tongxinluo Capsule* for Secondary Prevention After Acute Myocardial Infarction: A Systematic Review and Meta-Analysis of Randomized Controlled Trials

**DOI:** 10.3389/fphar.2018.00830

**Published:** 2018-08-03

**Authors:** Min Li, Chengyu Li, Shiqi Chen, Yang Sun, Jiayuan Hu, Chen Zhao, Ruijin Qiu, Xiaoyu Zhang, Qin Zhang, Guihua Tian, Hongcai Shang

**Affiliations:** ^1^Key laboratory of Chinese Internal Medicine of Ministry of Education and Beijing, Dongzhimen Hospital, Beijing University of Chinese Medicine, Beijing, China; ^2^School of Chinese Medicine, Hong Kong Baptist University, Hong Kong, Hong Kong; ^3^Institute of Integration of Traditional Chinese and Western Medicine, Guangzhou Medical University, Guangzhou, China

**Keywords:** *tongxinluo*, *TXL*, acute myocardial infarction, systematic review, meta-analysis

## Abstract

**Background:** Chinese patent medicine *Tongxinluo capsule* (*TXL*) is commonly used for cardio-cerebrovascular diseases. Previous research had demonstrated that *TXL* exhibited great clinical effects on the treatment of acute myocardial infarction (AMI), however there is a lack of systematic review. The purpose of this study was to evaluate the potential effectiveness and safety of *TXL* for secondary prevention in patients with AMI.

**Method:** We searched 6 databases to identify relevant randomized controlled trials (RCTs) from inceptions to December 30, 2017. Two review authors independently assessed the methodological quality and analyzed data by the RevMan 5.3 software. The publication bias was assessed through funnel plot and Begg's test. The Grading of Recommendations Assessment, Development and Evaluation (GRADE) approach was used for evaluating the quality of evidence.

**Results:** We included 19 RCTs in this review and performed a meta-analysis based on 16 studies. There were statistical differences of *TXL* treatment group in reducing primary cardiovascular events (cardiac death [RR = 0.27, 95%CI: 0.08~0.95, *I*^2^ = 0%], recurrent myocardial reinfarction [RR = 0.38, 95%CI: 0.20~0.74, *I*^2^ = 0%], arrhythmia [RR = 0.44, 95%CI: 0.30~0.66, *I*^2^ = 0%], recurrent angina pectoris [RR = 0.34, 95%CI: 0.17~0.69, *I*^2^ = 0%]). *TXL* could improve cardiac function (LVEF [MD = 4.10, 95%CI: 3.95~4.25, *I*^2^ = 0%]), regulate blood lipid TC [MD = −0.66, 95%CI: −0.94 ~ −0.37, *I*^2^ = 74%], TG [MD = −0.38, 95%CI: −0.62 ~ −0.14, I^2^ = 70%], LDL-C[−0.40, 95%CI: −0.65 ~ −0.16, *I*^2^ = 88%), decrease the level of hs-CRP (4-week: MD = −0.78, 95%CI: −0.97 ~ −0.60, *I*^2^ = 20%; Over 4-week: MD = −1.36, 95%CI: −1.55 ~ −1.17, *I*^2^ = 20%). However, *TXL* has little effects on revascularization [RR = 0.45, 95%CI: 0.13~1.56, *I*^2^ = 0%], recurrent heart failure (RR = 0.83, 95%CI: 0.27~2.57, *I*^2^ = 0%), and HDL-C (MD = 0.14, 95%CI: 0.00 ~0.29, *I*^2^ = 73%). Furthermore, *TXL* treatment group was more prone to suffer gastrointestinal discomfort.

**Conclusion:** Chinese patent medicine *TXL* seemed beneficial for secondary prevention after AMI. This potential benefit needs to be further assessed through more rigorous RCTs.

Systematic review registration number in the PROSPERO register: CRD42017068417.

## Introduction

Cardiovascular diseases (CVDs) are the major public health problem and a chief cause of morbidity and mortality around the world. Approximately 17.7 million people died from CVDs in 2015, accounting for 31% of all global deaths (WHO, 2017). Acute myocardial infarction (AMI) is the most severe type of CVD, causing more than 4 million deaths in Europe and northern Asia (Thygesen et al., 2012; Nichols et al., 2014). In United States, there would be an American diagnosed as myocardial infarction (MI) approximately every 40 s and the overall prevalence for MI was 3.0% in adults (Benjamin et al., 2017). In China, the number of CVD events manifestly increased from 0.75 million in 1990 to 1.4 million in 2013, and one million deaths were caused by MI annually (Li J. et al., 2015; Zhou et al., 2016). According to the National Institute of Health and Care Excellence clinical guideline, the treatment of AMI mainly contains primary percutaneous coronary intervention (PPCI), fibrinolysis, coronary angiography and so forth (Carville et al., 2015). And with the development of these revascularization treatments, the short-term prognosis of AMI has been greatly improved, but mortality and recurrence rate of patients with MI remain high after the acute phase of MI (García-García et al., 2010; Andrés et al., 2012). Secondary prevention of MI has been defined as a kind of evidence-based guideline recommendation and comprehensive intervention strategy to control various cardiovascular risk factors, reduce the recurrence of MI, heart failure (HF), arrhythmia, angina and the incidence of sudden death, extend patients' life and improve their life quality via using non-medical and medical therapy (Perk et al., 2013; Piepoli et al., 2018). Thus, it is vital to conduct secondary prevention for patients who suffered the MI as early as possible.

Nowadays, to decrease risk factors and mortality, available guidelines for secondary prevention of MI worldwide uniformly recommend interventions: one is the optimal use of cardio-protective medical therapies [aspirin, statins, beta-blockers and angiotensin converting enzyme inhibitors/ angiotensin receptor blockers (ACEI/ARBs)], the other one is non-medicine strategy, like smoking cessation and the increase of physical activities (Zeymer et al., 2011; Petersen et al., 2012; Bauters et al., 2014). Traditional Chinese medicine (TCM) has been used in patients with CVDs for thousands of years and is still being commonly used in modern times in both China and elsewhere worldwide (Tachjian et al., 2010). However, to date, it is still uncertain whether TCM can be used as a kind of drug therapy for secondary prevention of CVDs.

*Tongxinluo Capsule (TXL)*, passed appraisal by National Fund for new drug research and registered in the State Food and Drug Administration of China, is a formally classical Chinese patent medicine in treating unstable angina pectoris and acute coronary syndrome (Wang et al., 2003; Ma et al., 2009). The main ingredients of *TXL* consist of *Ginseng Radix Et Rhizoma (Ren Shen), Paeoniae Radix Rubra (Chi Shao), Ziziphi Spinosae Semen (Suan Zao Ren), Dalberglae Odoriferae Lignum (Jiang Xiang), Santali Albi Lignum (Tan Xiang), Olibanum (Ru Xiang), Hirudo (Shui Zhi), Scorpio (Quan Xie), Scolopendra (Wu Gong), Cicadae Periostracum (Chan Tui), Eupolyphaga Steleophaga (Tu Bie Chong)*, and *Borneolum Syntheticum (Bing Pian)*, which can supply qi, activate blood circulation, dredge collaterals and relieve pain according to the TCM theory (Pharmacopoeia, 2015). Besides, in clinical practice, TXL is taken orally 2-4 capsules per time, 3 times a day. The detailed production process and quality control of TXL was showed in Supplementary Material 1. Plenty of studies showed that *TXL* could enhance the stability of vulnerable plaques, inhibit inflammation, improve the left ventricular function and exhibit other pharmacological functions (Zhang L. et al., 2009; Zhang et al., 2010; Bai et al., 2013). A Cochrane systematic review indicated *TXL* could reduce the frequency of acute angina, improve electrocardiogram and angina symptoms (Wu et al., 2006). Currently, many studies reported *TXL* had good therapeutic effects on patients with MI, but there was little solid evidence about secondary prevention after AMI, thus, the purpose of this study was to perform a comprehensive review and meta-analysis of *TXL* for secondary prevention after AMI, especially for the primary cardiovascular events and heart functions.

## Methods

The review protocol was registered on PROSPERO (CRD42017068417), and the review was constructed following the PRISMA guidelines (Supplementary Material 2).

### Criteria for considering studies for this review

#### Types of studies

Randomized controlled trials (RCTs) with blind method or not and no limit of publishing language were included. The course of treatment was no less than 4–week, and the number of patients in treatment/control group was more than 30. We excluded the conference papers.

#### Types of participants

Patients diagnosed with AMI were included, not included those with severe liver and kidney dysfunction. Age and race were not limited.

#### Types of interventions

Based on the conventional western medicine treatment, the treatment group was given *TXL* alone or combined with western medicine, and the control group was given nothing, placebo or western medicine. According to clinical symptoms, the conventional western medicines are mainly for secondary prevention of MI, including aspirin (100 mg/d), clopidogrel (75 mg/d), metoprolol tartrate tablet (50 ~ 100 mg/bid), atorvastatin (20 mg/d) and so forth.

#### Types of outcome measures

##### Primary outcomes

①Primary cardiovascular events: cardiac death, recurrent myocardial infarction, revascularization, arrhythmia, heart failure and recurrent angina pectoris; ② Heart functions: left ventricular ejection fraction (LVEF). The included studies must contain the primary outcomes.

##### Secondary outcomes

①Lipid levels: total cholesterol (TC), triglyceride (TG), high density lipoprotein cholesterol (HDL-C), low-density lipoprotein cholesterol (LDL-C); ② Inflammatory response: hypersensitive C-reactive protein (hs-CRP); ③ Adverse events were calculated as follows: peripheral hemogram, liver function, renal function, gastrointestinal reactions and so forth.

### Search methods for identification of studies

The search was applied to PubMed, The Cochrane Library, Web of Science, China National Knowledge Infrastructure (CNKI), Wanfang Database, and Chinese Scientific Journal Database (VIP) from inception to December 30, 2017. The search strategy used the following general terms individually or combined: “tong-xin-luo,” “Tong-xin-luo,” “Tong xin luo,” “tong xin luo,” “Tongxinluo,” “tongxinluo,” “myocardial infarct,^*^” “heart attack,^*^” “heart disease,^*^” “ischemia reperfusion,” “patient.^*^” The detail search strategy was shown in Supplementary Material 3.

### Data extraction and management

#### Data extraction and management

Two investigators (QZ and JYH) independently conducted the literature searching, study selection, and data extraction. The extracted data of included studies was filled in a standardized form prepared for this review. The extracted data included the first author, year of publication, disease type, sample size, randomized method, average age of participants, treatment duration, interventions in the treatment and control group, dose of medicines, outcomes and so on. Disagreements were discussed and resolved in a consensus meeting with the corresponding author (HCS). If the literature has multiple time end indicators, we chose the longest one.

#### Unit of analysis issues

Subgroups were divided according to intervention or course of treatment and analyzed individually.

#### Assessment of quality of included studies

According to Cochrane Reviewer's Handbook, two of the authors (ML and RJQ) individually assessed the risk of bias. Six evaluation criteria of the quality of RCTs were used, which involved the generation of random sequence, randomization concealment, blind method, integrity of outcome data, selective reporting and other bias.

#### Measures of treatment effect

Revman 5.3 software provided by the Cochrane Collaboration was used for data analyses. Continuous outcomes were presented as weighted mean difference (WMD), dichotomous data as risk ratio (RR) and 95% confidence interval (CI). Means and standard deviations were extracted for continuous outcomes, and as for the dichotomous data, we recorded the number of patients who experienced the event in each group.

#### Assessment of heterogeneity

Clinical heterogeneity of included studies was analyzed with χ^2^ test. If *I*^2^ < 50%, then there is no statistical heterogeneity between studies, and fixed effect model was used for data analysis; If *I*^2^ > 50%, then statistical heterogeneity between studies exists, random effect model was used, and the cause of heterogeneity was analyzed. Subgroup analysis was used when clinical heterogeneity exists. Subgroup was divided according to the sources of heterogeneity, such as intervention measure, course, and dosage of medicines. Descriptive analysis was used if clinical heterogeneity still exists.

#### Assessment of publication biases

The potential publication bias was assessed using funnel plots and Begg's test by Stata 14 software (Begg and Mazumdar, 1994). If *P* > 0.05, there is no publication bias, whereas publication bias exists.

#### Sensitivity analysis

Compare the pooled statistics before and after excluding studies of low quality and great weight and those which have different results from other studies. If they have the same results (both results have differences or have no differences), then the meta-analysis result is stable and vice versa.

### Statistical analysis

The primary and second outcomes from the included studies were entered into Revman 5.3 (Higgins and Green, 2011). *P* < 0.05 was considered statistically significant.

### Grade

The Grading of Recommendations Assessment, Development and Evaluation (GRADE) approach was used to evaluate the quality of evidence by ML and XYZ, which was classified as high, moderate, low, or very low based on the judgment of the risk of bias, inconsistency, indirectness, imprecision and other considerations (Schunemann et al., 2013). Summary of Findings (SOF) tables were prepared using the software program “GRADE pro GDT.”

## Results

### Description of included trials

#### Search process

**Figure 1:**
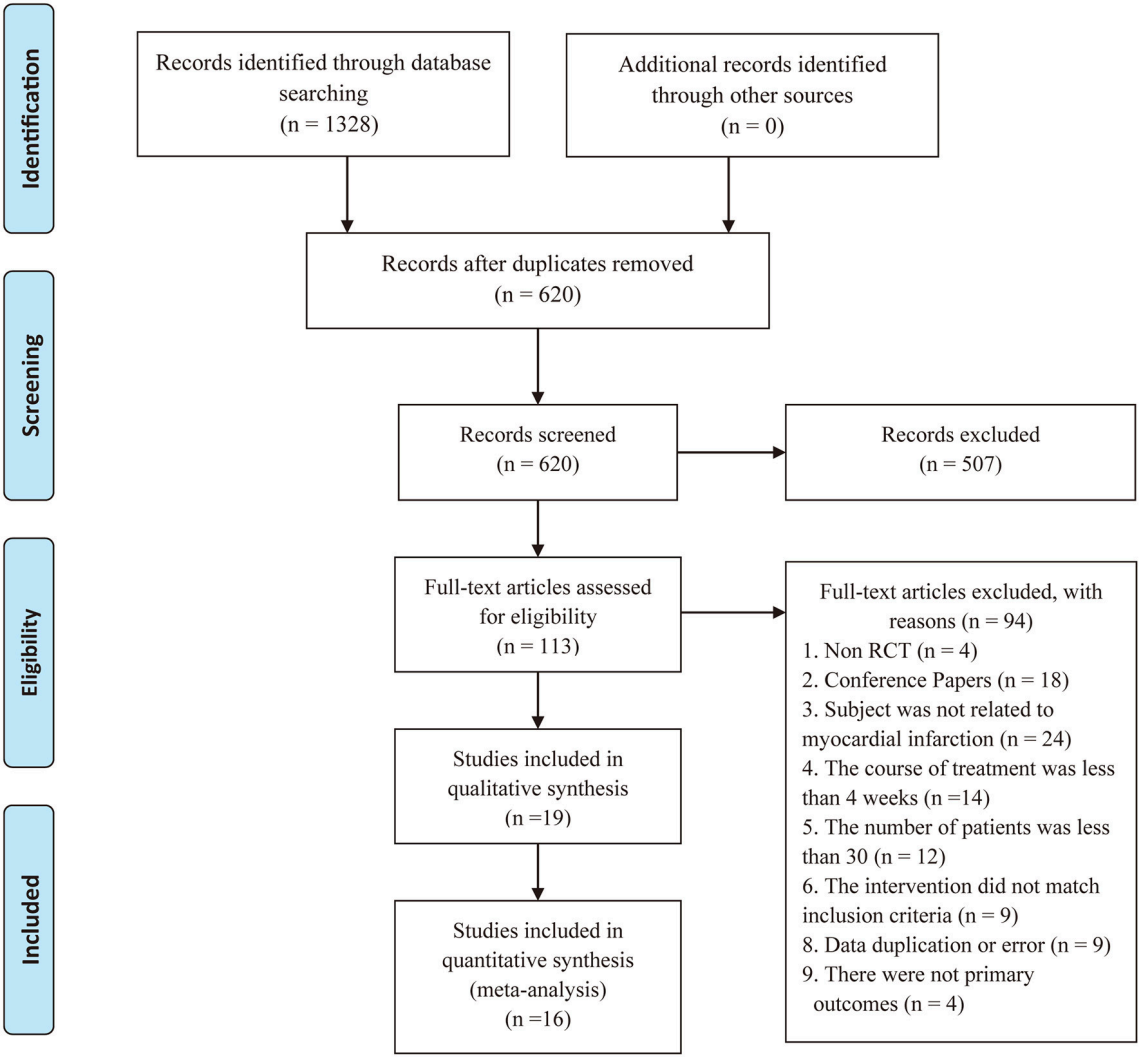
Process of searching and screening studies.

The initial search using the electronic search strategies yielded 1328 studies. After removing 708 duplicates from different databases, we evaluated 620 potentially relevant articles for eligibility. After screening the titles and abstracts, we excluded 507 studies. Of the 113 remaining studies, we further excluded 94 studies after screening the full-text articles. Eventually, we included 19 studies. There may exist the clinical heterogeneity between long-term intervention (≥1–year) and short-term duration (< 1–year) in included studies. Thus, we conducted a descriptive analysis of 3 articles (Zhang and Zhang, 2009; Shen, 2011; Wang Y. L. et al., 2016) with long-term intervention and do a meta-analysis of the remaining 16 studies (You et al., 2005, 2012; Chen et al., 2007, 2008; Huang, 2010; Liang et al., 2010; Liao et al., 2010; Tian and Li, 2011; Yang and Yu, 2011; Tian et al., 2014; Qin, 2015; Xia et al., 2016; Fan et al., 2017; Li X. F. et al., 2017; Peng et al., 2017; Wu, 2017). A flow chart (Figure 1) illustrates our search process and study selection.

#### Included studies

We included 19 studies in this review. All studies were conducted in China and published in full.

#### Participants

Total of 1877 participants with AMI were included. The average sample size of the trials was 99 participants (ranging from 60 to 190 participants per trial). The course of treatment fluctuated between 4–week and 2–year.

#### Interventions

Based on the conventional western medicine treatment, there were 12 studies (You et al., 2005, 2012; Chen et al., 2007; Zhang and Zhang, 2009; Huang, 2010; Liang et al., 2010; Liao et al., 2010; Tian et al., 2014; Qin, 2015; Wang Y. L. et al., 2016; Xia et al., 2016; Peng et al., 2017) for *TXL* vs. blank control. 6 studies (Shen, 2011; Tian and Li, 2011; Yang and Yu, 2011; Fan et al., 2017; Li X. F. et al., 2017; Wu, 2017) made a comparison between *TXL* plus western medicine and western medicine. One study (Chen et al., 2008) showed *TXL* versus placebo. Further details were given in Table 1.

**Table 1:** The detailed information of included studies.

**Studies**	**Disease**	**Sample (T/C)**	**Age (T/C)**	**Interventions (T/C)**	**Duration**	**Outcomes**
Fan et al., 2017	STEMI+PCI	95/95	32~73/34~74	*TXL* (3 capsules/tid) + Trimetazidine (20 mg/tid) +CWMT/ Trimetazidine (20 mg/tid) + CWMT	12-week	⑦ ⑫
Li X. F. et al., 2017	STEMI+PCI	50/50	70 ± 9/72 ± 8	*TXL* (3 capsules/tid) + Trimetazidine (20 mg/tid) +CWMT/Trimetazidine (20 mg/tid) + CWMT	12-week	⑦
Peng et al., 2017	AMI+PCI	85/85	61.3 ± 3.8/62.8 ± 3.5	*TXL* (4 capsules/tid) + CWMT/ CWMT	6-month	①②③⑥
Wu, 2017	STEMI+PCI	30/30	65.03 ± 8.81/63.23 ± 9.50	*TXL* (4 capsules/tid) + Atorvastatin Calcium (10 mg/d) + CWMT/ Atorvastatin Calcium (10 mg/d) + CWMT	4-week	①②⑦⑧⑨⑩ ⑪ ⑫ ⑬
Wang Y. L. et al., 2016	AMI+PCI	30/30	58 ± 7/58 ± 6	*TXL* (4 capsules/tid) + CWMT/CWMT	1-year	⑦
Xia et al., 2016	AMI+PCI	37/37	51.5 ± 13.5/51.5 ± 15.2	*TXL* (4 capsules/tid) + CWMT/CWMT	4-week	② ⑫
Qin, 2015	STEMI+PCI	50/50	56.5 ± 11.2/58.1 ± 11.4	*TXL* (4 capsules/tid) + CWMT/CWMT	60-day	①②④⑤⑥⑦⑧⑨⑩ ⑪
Tian et al., 2014	AMI+PCI	30/30	54.9 ± 10.4/54.5 ± 9.8	*TXL* (4 capsules/tid) + CWMT/CWMT	3-month	①②③⑥⑦ ⑫
You et al., 2012	AMI	45/46	46 ± 8.6/47 ± 5.2	*TXL* (4 capsules/tid) + CWMT/CWMT	8-week	⑦
Yang and Yu, 2011	AMI	42/34	62.48 ± 10.05/63.37 ± 9.27	*TXL* (4 capsules/tid) + Metoprolol sustained release tablets (23.75–47.5 mg/d) + CWMT/Metoprolol tablets (6.26-12.5 mg/d) +CWMT	4-week	④ ⑬
Tian and Li, 2011	AMI+ thrombolysis	51/51	57 ± 12/61 ± 11	*TXL* (4 capsules/tid) + Urokinase + CWMT/Urokinase + CWMT	4-week	④⑦ ⑬
Shen, 2011	AMI+ thrombolysis	51/47	62.87 ± 3.65/63.21 ± 3.92	*TXL* (3 capsules/tid) + Simvastatin (80 mg/d) + CWMT/ Simvastatin (20 mg/d) + CWMT	1-year	①③ ⑪ ⑬
Liao et al., 2010	AMI	39/37	60.3 ± 9.9/64.3 ±11.7	*TXL* (3–4 capsules/tid) + CWMT/CWMT	4-week	⑦ ⑫
Huang, 2010	STEMI+PCI	62/58	58.3 ± 12.6	*TXL* (4 capsules/tid) + CWMT/CWMT	6-month	①②③⑧⑨ ⑫ ⑬
Liang et al., 2010	AMI+PCI	42/38	40~70	*TXL* (2 capsules/tid) + CWMT/CWMT	6-month	②⑥⑧⑨⑩ ⑪
Zhang and Zhang, 2009	AMI+PCI	96/82	43~70	*TXL* (3 capsules/tid) + CWMT/CWMT	24-month	①②③⑤⑥ ⑬
Chen et al., 2008	AMI	35/35	68 ± 7/69 ± 7	*TXL* (3 capsules/tid) + CWMT/Placebo (3 capsules/tid) + CWMT	6-week	⑦
Chen et al., 2007	AMI	30/30	58 ± 11	*TXL* (2-4 capsules/tid) + CWMT/CWMT	8-week	①②④⑤⑥⑦⑧⑨⑩ ⑪ ⑬
You et al., 2005	STEMI+PCI	60/52	Unclear	*TXL* (4 capsules/tid) + CWMT/CWMT	6-month	⑦

*PCI, percutaneous coronary intervention; STEMI, ST-segment elevation myocardial infarction; CWMT, Conventional western medicine treatment; Tid, Three times a day; Qd, One time a day; ① Cardiac death; ② Recurrent myocardial infarction; ③ Revascularization; ④ Arrhythmia; ⑤ Heart failure; ⑥ Recurrent angina pectoris; ⑦ LVEF; ⑧ TC; ⑨ TG; ⑩ HDL-C; ⑪ LDL-C; ⑫ hs-CRP; ⑬ Adverse reactions*.

#### Risk of bias assessment

**Figure 2:**
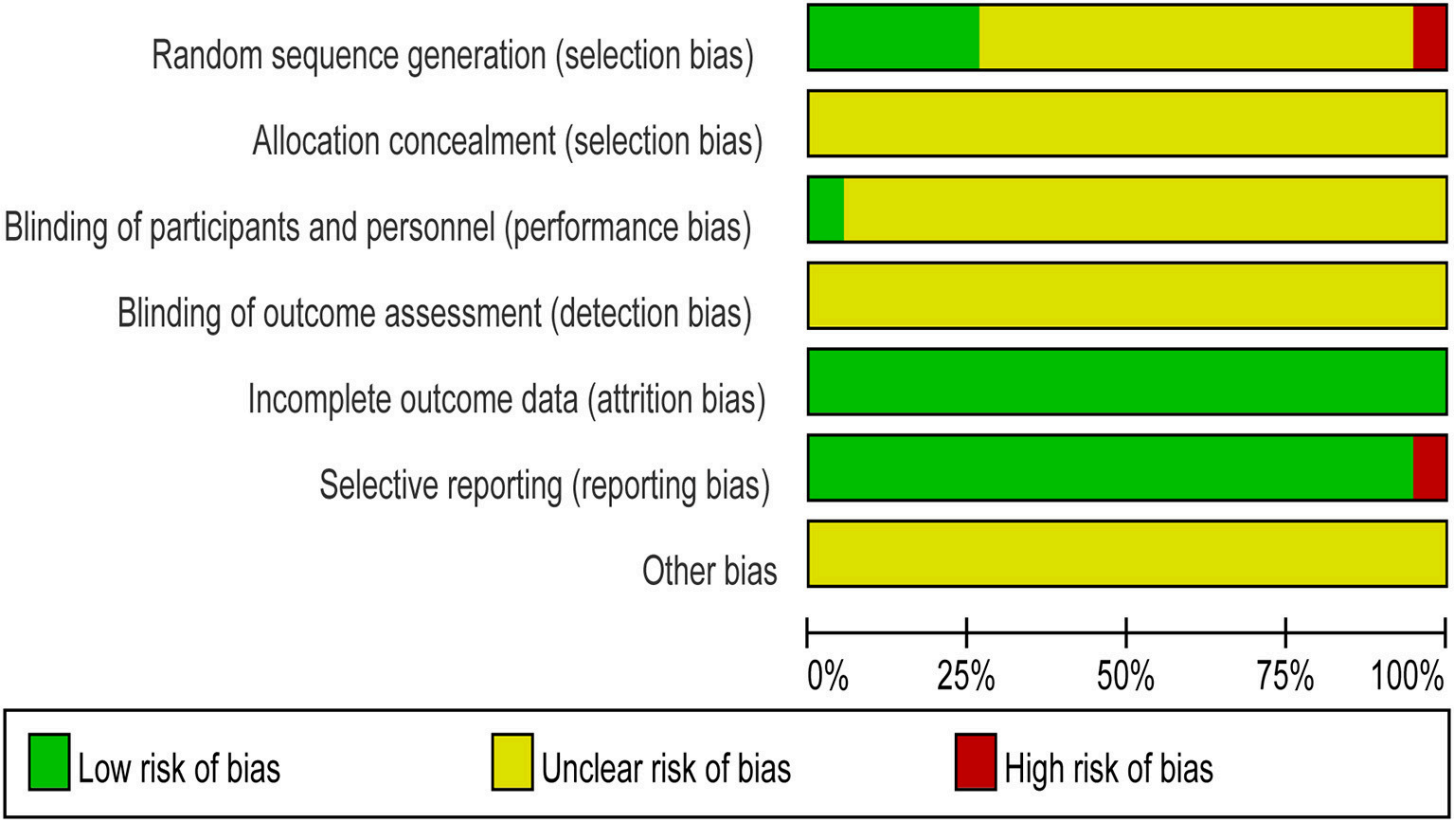
Risk of bias graph.

All the trials provided very limited information about design and methodology. Six studies (Chen et al., 2008; Zhang and Zhang, 2009; Tian et al., 2014; Qin, 2015; Xia et al., 2016; Wu, 2017) described the random sequence generation methods, among which 5 articles (Chen et al., 2008; Tian et al., 2014; Qin, 2015; Xia et al., 2016; Wu, 2017) showed the random number table, the other one used lottery (Zhang and Zhang, 2009). The rest included studies only mentioned random, did not reported it in detail. In addition, allocation concealment and blind were not very clear. Only one study used a double-blind method (Chen et al., 2008). None of the trials had a pretrial estimation of sample size, which indicated the lack of statistical power to ensure appropriate estimation of the therapeutic effect, as shown in Figure 2.

### Effects of interventions

We conducted a descriptive analysis of 3 studies. A study (Wang Y. L. et al., 2016) showed TXL could increase LVEF (*P* < 0.05), and the other 2 articles mainly reported the role of TXL on primary cardiovascular events. One study (Shen, 2011) exhibited TXL was beneficial to reduce the total cardiovascular events, the other (Zhang and Zhang, 2009) demonstrated TXL had no effects on cardiac death, non-fatal MI, HF, recurrent angina pectoris except revascularization. Then we did a meta-analysis of the remaining 16 studies with a short-term duration and assessed the clinical functions of TXL by primary/s outcomes.

#### Primary outcomes

##### Primary cardiovascular events

**Figure 3:**
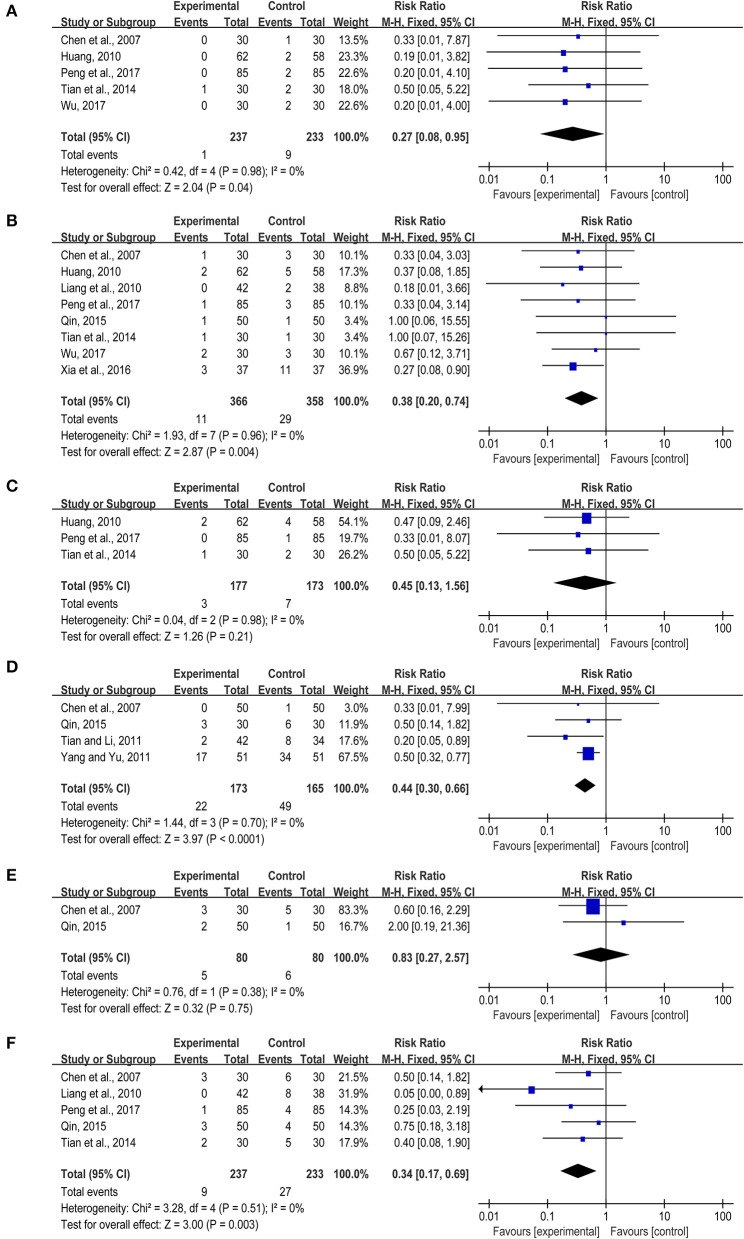
**(A–F)** Primary cardiovascular events.

Based on the western conventional treatments, *TXL* alone or combined with other western medicine could lower the risk of cardiovascular events effectively. Six studies (Chen et al., 2007; Huang, 2010; Tian et al., 2014; Qin, 2015; Peng et al., 2017; Wu, 2017), including 470 patients, reported cardiac death. One of the researches (Qin, 2015) showed no death in either treatment group or control group, so we conducted the meta-analysis on the other 5 studies (Chen et al., 2007; Huang, 2010; Tian et al., 2014; Peng et al., 2017; Wu, 2017), indicating that *TXL* had better efficacy on lowering the cardiac death rate compared with the control group (RR = 0.27, 95% CI: 0.08~0.95, *I*^2^ = 0%) (Figure 3A). Eight articles (Chen et al., 2007; Huang, 2010; Liang et al., 2010; Tian et al., 2014; Qin, 2015; Xia et al., 2016; Peng et al., 2017; Wu, 2017) reported the recurrent myocardial infarction, 11 cases happened in treatment group while 29 cases occurred in control group, showing that *TXL* had the lower rate of reinfarction (RR = 0.38, 95%CI: 0.20~0.74, *I*^2^ = 0%) (Figure 3B). Three studies revealed the revascularization of patients with myocardial infarction (Huang, 2010; Tian et al., 2014; Peng et al., 2017), there was no evidence that the treatment group was better than the control group (RR = 0.45, 95%CI: 0.13~1.56, *I*^2^ = 0%) (Figure 3C). Besides, *TXL* presented the less cardiac arrhythmia (RR = 0.44, 95%CI: 0.30~0.66, *I*^2^ = 0%) (Chen et al., 2007; Tian and Li, 2011; Yang and Yu, 2011; Qin, 2015) (Figure 3D). Eleven patients suffered from heart failure in 2 studies (Chen et al., 2007; Qin, 2015), the number of treatment group and control group was 5 and 6, respectively. There was no statistical significance in heart failure (RR = 0.83, 95%CI: 0.27~2.57, *I*^2^ = 0%) (Figure 3E). In addition, 5 articles (Chen et al., 2007; Liang et al., 2010; Tian et al., 2014; Qin, 2015; Peng et al., 2017) reported recurrent angina pectoris, involving in 470 participants, indicating *TXL* could markedly lower relapse rate than the control group (RR = 0.34, 95%CI: 0.17~0.69, *I*^2^ = 0%) (Figure 3F).

##### Heart function

**Figure 4:**
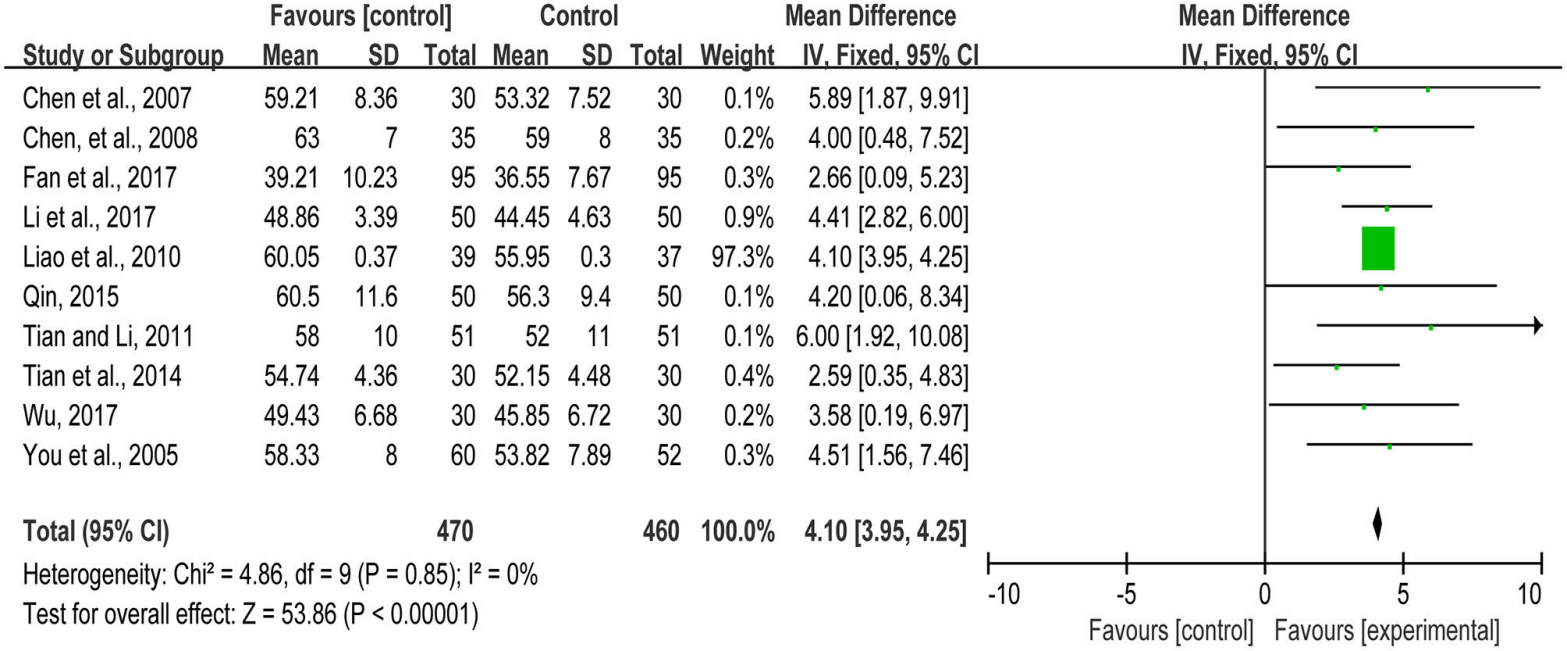
The LVEF of included studies.

AMI is the most severe manifestation of coronary artery disease, which has a negative effect on the myocardial function and blood supply. LVEF is closely related to the myocardial contractility, reflecting myocardial systolic function and bloodstream of the coronary arteries. Eleven studies (You et al., 2005, 2012; Chen et al., 2007, 2008; Liao et al., 2010; Tian and Li, 2011; Tian et al., 2014; Qin, 2015; Fan et al., 2017; Li X. F. et al., 2017; Wu, 2017) reported the LVEF of the patients with myocardial infarction. While there was one study (You et al., 2012) detecting LVEF by radionuclide ventriculography, others used the doppler echocardiography. Thus, the meta-analysis we conducted from the remaining ten studies. The meta-analysis showed that TXL treatment group could increase the LVEF significantly and improve heart function (MD = 4.10, 95%CI: 3.95~4.25, *I*^2^ = 0%) (Figure 4).

#### Secondary outcomes

##### The blood lipid levels

**Figure 5:**
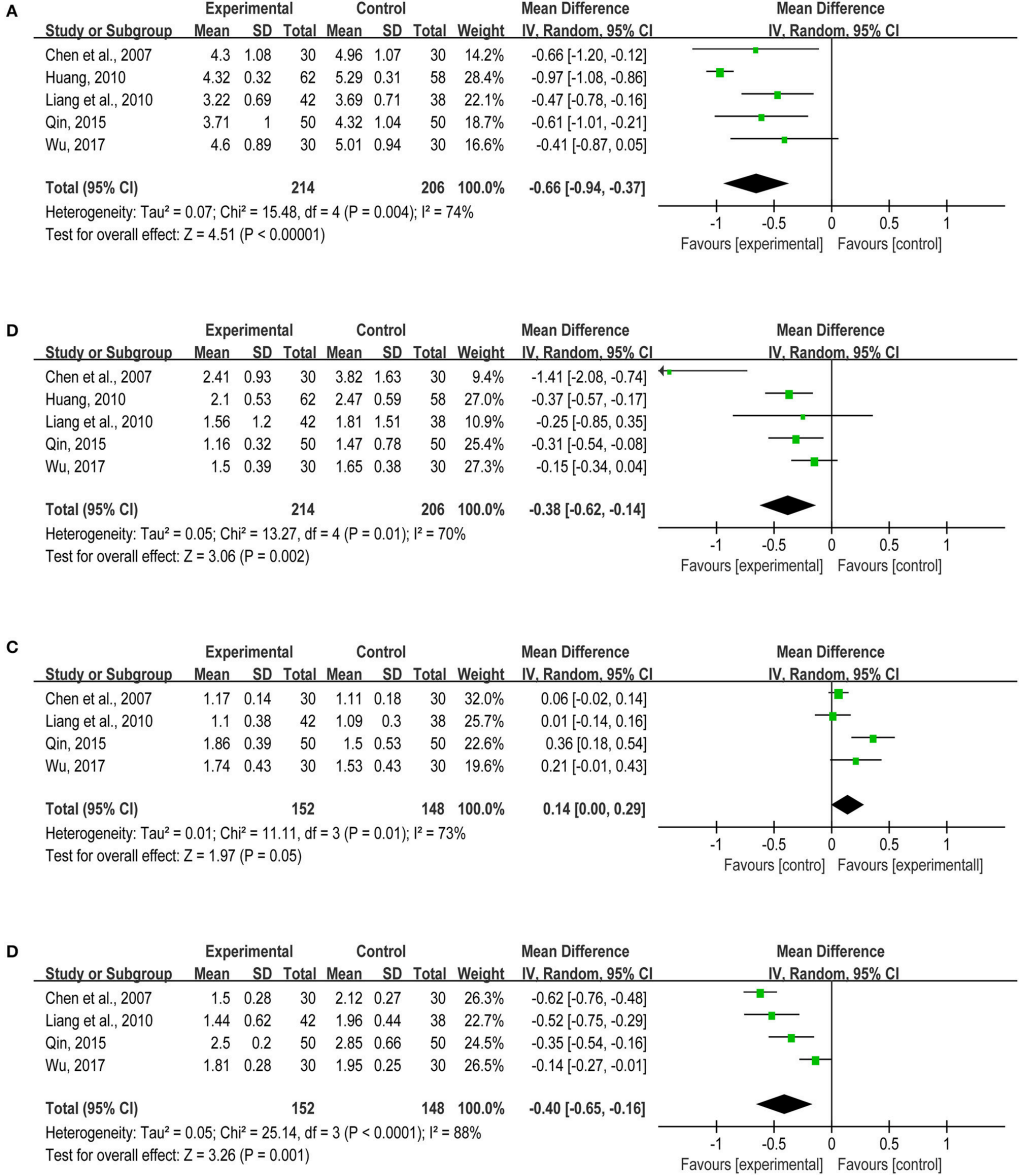
**(A–D)** The blood lipid level.

**Figure 6:**
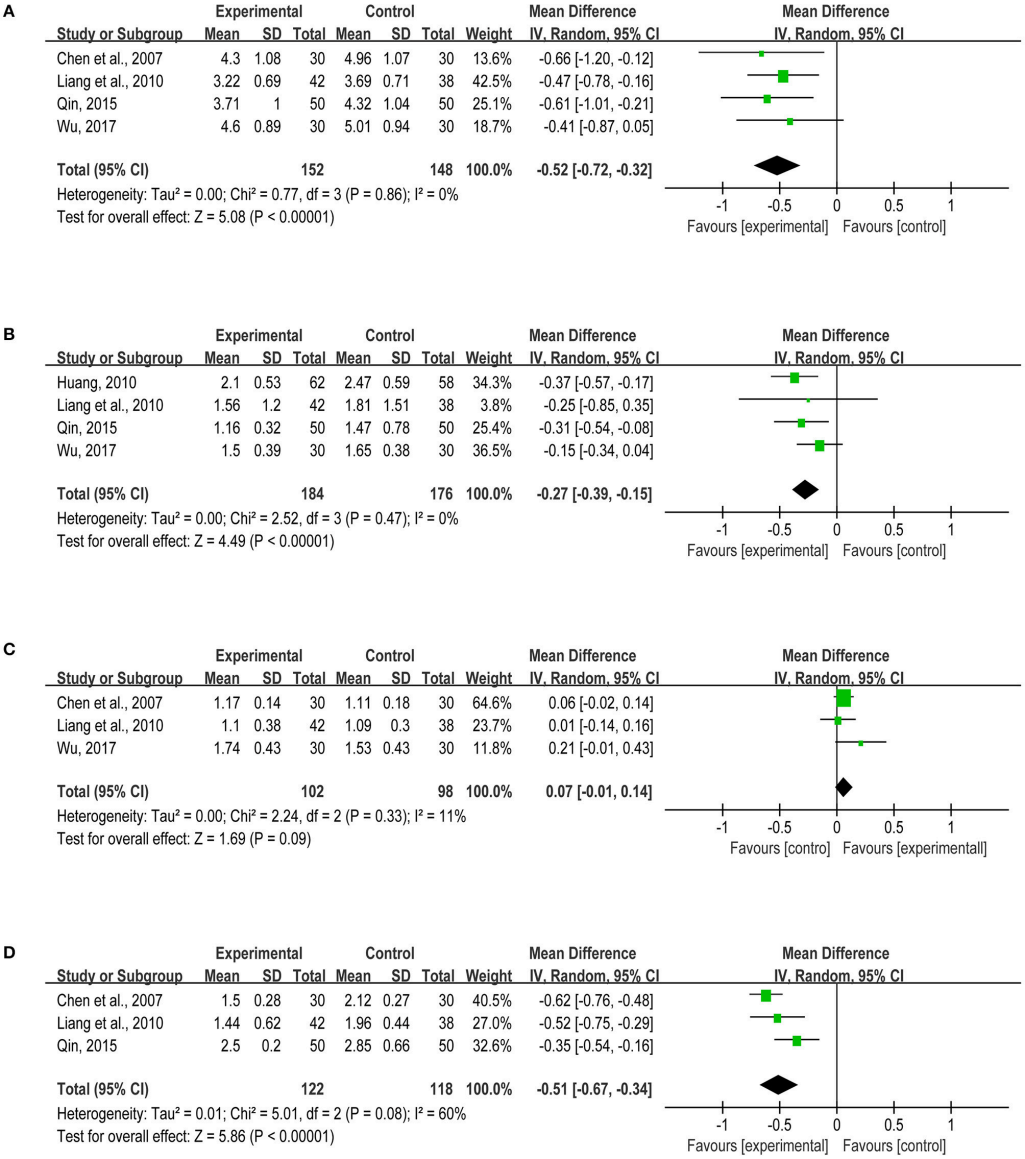
**(A–D)** The blood lipid level (sensitivity analysis).

Atherosclerosis is the main pathogenesis of myocardial infarction in coronary heart disease, which is affected by many factors, such as lipid metabolism disorder. Current researches indicated that dyslipidemia is one of the risk factors causing atherosclerosis, myocardial infarction and other vascular diseases (Cheng et al., 2015; Górski et al., 2016). On the basis of statin therapy in both treatment and control groups, the included studies indicated that *TXL* could effectively regulate lipid metabolism. Five articles (Chen et al., 2007; Huang, 2010; Liang et al., 2010; Qin, 2015; Wu, 2017) reported TC and TG, including 214 patients in the treatment group and 206 sufferers in the control group. *TXL* could remarkably reduce the level of TC (MD = −0.66, 95%CI: −0.94 ~ −0.37, *I*^2^ = 74%) and TG (MD = −0.38, 95%CI: −0.62 ~ −0.14, I^2^ = 70%) (Figures 5A,B). Due to the large heterogeneity, the sensitivity analysis of TC was performed. When we removed one literature (Huang, 2010), the result did not change significantly (MD = −0.52, 95%CI: −0.72 ~ −0.32, *I*^2^ = 0%) (Figure 6A). The sensitivity analysis of TG presented the similar outcome after eliminating one study (Chen et al., 2007) (MD = −0.27, 95%CI: −0.39~-0.15, *I*^2^ = 0%) (Figure 6B). A low level of plasma HDL-C is a strong and independent risk factor for atherosclerotic cardiovascular disease. There was no statistical significance in HDL-C from four studies (Chen et al., 2007; Liang et al., 2010; Qin, 2015; Wu, 2017) (MD = 0.14, 95%CI: 0.00 ~0.29, *I*^2^ = 73%) (Figure 5C), and such difference between treatment group and control group was unchanged through sensitivity analysis when we removed one study (Qin, 2015) (MD = 0.07, 95%CI: −0.01~0.14, *I*^2^ = 11%) (Figure 6C). Moreover, according to the extracted data from four articles (Chen et al., 2007; Liang et al., 2010; Qin, 2015; Wu, 2017), *TXL* enabled to lower LDL-C significantly (MD = −0.40, 95%CI: −0.65 ~ −0.16, *I*^2^ = 88%) (Figure 5D). The sensitivity analysis of LDL-C presented the similar result after eliminating one study (Wu, 2017) (MD = −0.51, 95%CI: −0.67 ~ −0.34, *I*^2^ = 60%) (Figure 6D).

##### The inflammation reaction

**Figure 7:**
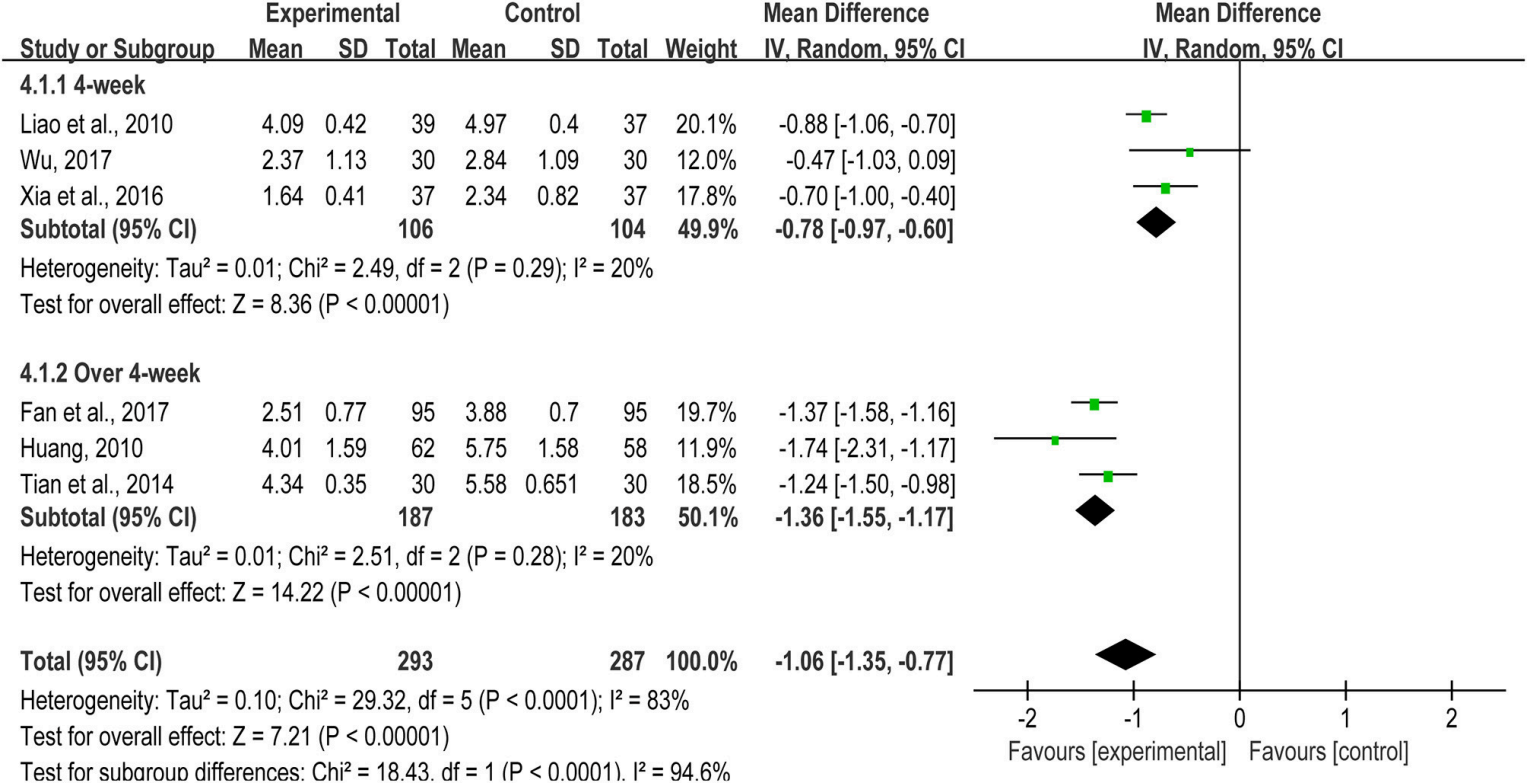
The hs-CRP of included studies.

Inflammation reaction is one of the key links of AMI progression. The necrosis of numerous cardiomyocytes in the infarct tissue will lead to a sharp inflammation. The rapid accumulating of massive leukocytes and monocytes accompanied by the release of a great number of inflammatory factors, leading to further aggravate myocardial injury (Stirrat et al., 2017). Hs-CRP, one kind of C-reactive protein in plasma, is a non-specific marker of systemic inflammatory response in acute phase, could predict the risk of cardiovascular events. Six studies (Huang, 2010; Liao et al., 2010; Tian et al., 2014; Xia et al., 2016; Fan et al., 2017; Wu, 2017) revealed hs-CRP involving in 293/287 patients in both groups. Due to high heterogeneity, we carried out a subgroup analysis based on different treatment courses. When the intervention time was 4 weeks, TXL treatment group could effectively reduce the hs-CRP level (MD = −0.78, 95%CI: −0.97 ~ −0.06, *I*^2^ = 20%), and this therapeutic effect would get better along with the prolonging of treatment period (MD = −1.36, 95%CI: −1.55 ~ −1.17, *I*^2^ = 20%) (Figure 7).

### Adverse reaction

Seven studies (Chen et al., 2007; Zhang and Zhang, 2009; Huang, 2010; Shen, 2011; Tian and Li, 2011; Yang and Yu, 2011; Wu, 2017), involving in 362/332 patients in two groups, have reported the adverse events of the TXL. Adverse drug reactions mainly included the gastrointestinal discomfort, transaminase elevation, stroke, pulmonary infection and so forth. Compared with the control group, the total risk rate of adverse events in the treatment group was relatively high (6.91% versus 4.22%), and the gastrointestinal discomfort accounted for the main proportion, as shown in Table 2.

**Table 2:** The adverse reaction of seven included studies.

**Adverse events**	**Treatment group (362)**	**Control group (332)**
Gastrointestinal discomfort	16	3
Transaminase elevation	6	5
Stroke	0	1
Hypotension	1	1
Pulmonary infection	0	1
Itch of skin	2	2
Allergic reaction	0	1
Total risk rate (%)	6.91	4.22

### Publication biases

**Figure 8:**
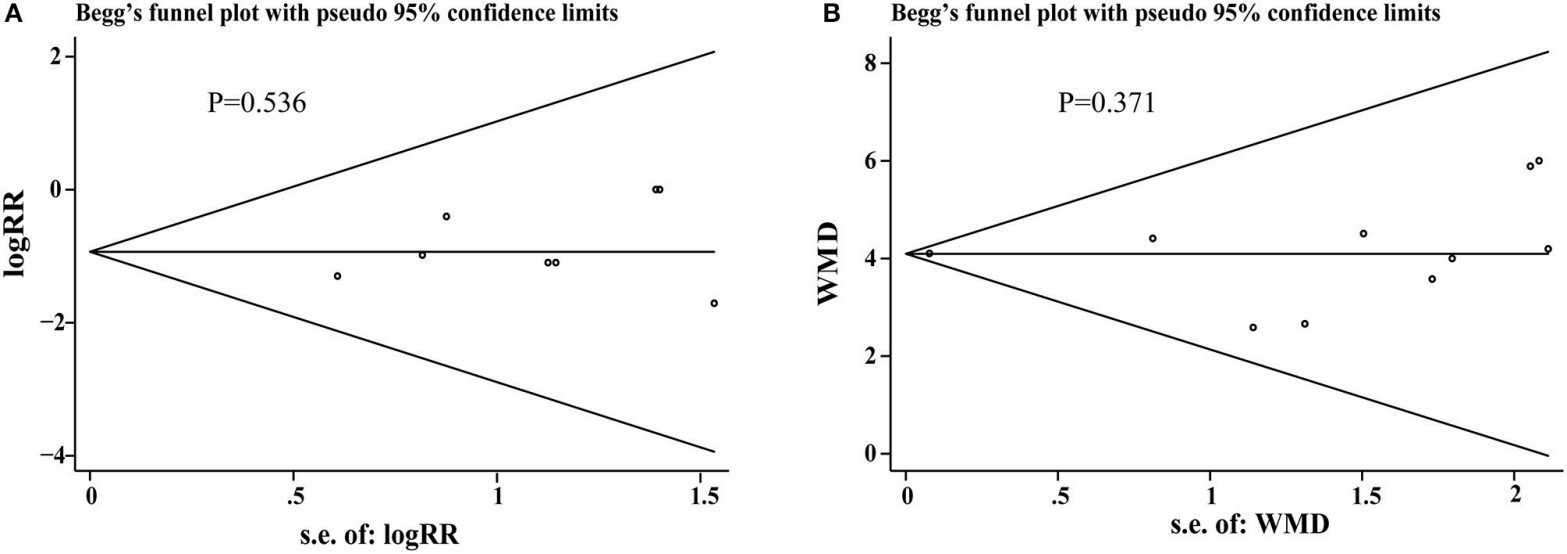
**(A,B)** The publication bias.

We proceed the publication bias (Recurrent myocardial reinfarction and LVEF) to evaluate the quality of our meta-analysis by funnel plot and Begg' test using State 14 software. Funnel chart analysis showed there were no obvious publication biases and *P*-values were 0.536, 0.371, respectively, as shown in Figures 8A,B.

### Grade

The quality of evidence was evaluated by ML and XYZ with GRADE approach, and most of cardiac events were considered as the moderate score. Some outcomes exhibited the low or very low evidence due to the large heterogeneity and/or the wide confidence interval (Table 3).

**Table 3:** GRADE quality of evidence summary table.

**Outcomes**	Illustrative comparative risks^*^ **(95% CI)**		**Relative effect (95% CI)**	**No of Participants (studies)**	**Quality of the evidence (GRADE)**
	**Assumed risk**	**Corresponding risk**			
	**Control group**	**Treatment group**			
Cardiac death	Study population		RR 0.27 (0.08 to 0.95)	470 (5 studies)	⊕⊕⊕⊖ moderate^a^
	39 per 1000	10 per 1000 (3–37)			
	Moderate				
	35 per 1000	9 per 1000 (3–33)			
Myocardial reinfarction	Study population		RR 0.38 (0.2–0.74)	724 (8 studies)	⊕⊕⊕⊖ moderate^a^
	81 per 1000	31 per 1000 (16–60)			
	Moderate				
	69 per 1000	26 per 1000 (14–51)			
Revascularization	Study population		RR 0.45 (0.13–1.56)	350 (3 studies)	⊕⊕⊕⊖ moderate^a^
	40 per 1000	18 per 1000 (5–63)			
	Moderate				
	67 per 1000	30 per 1000 (9–105)			
Arrhythmia	Study population		RR 0.44 (0.3–0.66)	338 (4 studies)	⊕⊕⊕⊖ moderate^a^
	297 per 1000	131 per 1000 (89–196)			
	Moderate				
	218 per 1000	96 per 1000 (65–144)			
Heart failure	Study population		RR 0.83 (0.27–2.57)	160 (2 studies)	⊕⊕⊖⊖ low^a^^,^^b^
	75 per 1000	62 per 1000 (20–193)			
	Moderate				
	93 per 1000	77 per 1000 (25–239)			
Recurrent angina pectoris	Study population		RR 0.34 (0.17–0.69)	470 (5 studies)	⊕⊕⊕⊖ moderate^a^
	116 per 1000	39 per 1000 (20–80)			
	Moderate				
	167 per 1000	57 per 1000 (28–115)			
LVEF	The mean lvef in the intervention groups was 4.1 higher (3.95–4.25 higher)			930 (10 studies)	⊕⊕⊕⊖ moderate^a^
TC	The mean tc in the intervention groups was 0.66 lower (0.94–0.37 lower)			420 (5 studies)	⊕⊕⊖⊖ low^a^^,^^c^
TG	The mean tg in the intervention groups was 0.38 lower (0.62–0.14 lower)			420 (5 studies)	⊕⊕⊖⊖ low^a^^,^^d^
HDL	The mean hdl in the intervention groups was 0.14 higher (0–0.29 higher)			300 (4 studies)	⊕⊖⊖⊖ very low^a^^,^^b^^,^^e^
LDL	The mean ldl in the intervention groups was 0.4 lower (0.65–0.16 lower)			300 (4 studies)	⊕⊕⊕⊖ moderate^a^^,^^f^
hs-CRP	The mean hs-crp in the intervention groups was 1.06 lower (1.35–0.77 lower)			580 (6 studies)	⊕⊖⊖⊖ very low^a^^,^^g^

* *The basis for the assumed risk (e.g., the median control group risk across studies) is provided in footnotes. The corresponding risk (and its 95% confidence interval) is based on the assumed risk in the comparison group and the relative effect of the intervention (and its 95% CI)*.

*CI, Confidence interval; RR, Risk ratio*.

*GRADE Working Group grades of evidence*.

*High quality, Further research is very unlikely to change our confidence in the estimate of effect*.

*Moderate quality, Further research is likely to have an important impact on our confidence in the estimate of effect and may change the estimate*.

*Low quality, Further research is very likely to have an important impact on our confidence in the estimate of effect and is likely to change the estimate*.

*Very low quality, We are very uncertain about the estimate*.

a *The random and blind of some studies were not clear*.

b *The confidence interval was wide*.

c *I^2^ of 74% showed the inconsistence among studies*.

d *I^2^ of 70% showed the inconsistence among studies*.

e *I^2^ of 73% showed the inconsistence among studies*.

f *I^2^ of 88% showed the inconsistence among studies*.

g *I^2^ of 83% showed the inconsistence among studies*.

## Discussion

In this meta-analysis from 16 studies of *TXL*, we observed the results as following: There was significant difference of *TXL* in preventing restenosis and recurrence of cardiovascular events (cardiac death, myocardial reinfarction, arrhythmia, recurrent angina pectoris). *TXL* enabled to improve cardiac function (LVEF), regulate lipid metabolism (TC, TG, LDL), and inhibit inflammation reaction (hs-CRP). However, *TXL* has little effects on revascularization, recurrent heart failure, and HDL. Moreover, *TXL* treatment group was more prone to suffer gastrointestinal discomfort.

### Effectiveness of *TXL*

AMI is a common cardiovascular disease, remaining a leading cause of morbidity and mortality worldwide. As we all know, AMI is caused by atherosclerotic coronary artery stenosis, plaque rupture and thrombosis appear, engendering insufficient blood supply of coronary, further resulting in myocardial ischemia or even necrosis. If AMI does not promptly recanalize the infarct-related coronary arteries after the onset of the disease, many patients eventually die of ventricular arrhythmias and heart failure. Despite substantial improvements in prognosis over the past decade, the progress is a result of several major trends, including improvements in risk stratification, more widespread use of an invasive strategy and medication (Kvakkestad et al., 2016). However, mortality and recurrence rate of the adverse cardiac events remain high after the acute phase of MI which will seriously affect the patients' quality of life and prognosis. Therefore, the second prevention of adverse cardiac events after MI is very important. Coronary artery in patients with MI has extensive lesions and multiple “vulnerable” plaques. While interventional therapy is often focusing on some single serious plaques, it is vital for many drugs to widely protect cardiovascular system through inhibiting vascular inflammation, improving myocardial energy metabolism, reducing myocardial remodeling, regulating blood lipids and so forth. Compared with the control group, *TXL* alone or combined with conventional western medicine exhibited a specific advantage in the secondary prevention of AMI, likely due to the comprehensive protection of blood vessels and myocardium. Many clinical trials and experimental evidence have just proved it.

#### Endothelial protective effect

Dysfunction of endothelial cells is directly associated with impaired vasorelaxation, increased inflammation, and increased migration and proliferation of smooth muscle cells, is the initiating factor in CVDs (Gutiérrez et al., 2013). And oxidized low-density lipoprotein- (ox-LDL-) induced dysfunction of the vascular endothelium is one of the key factors in the pathogenesis of many CVDs (Wang et al., 2009). It has been demonstrated that *TXL* alleviated ox-LDL-induced hyperpermeability via the activation of ERK1/2 in the vascular endothelial cell (Chang et al., 2017). Vascular injury after chronic hypoxia usually leads to endothelial injury and structural damage to tight junctions, *TXL* could promote endothelial cell proliferation and reverse the expression of tight junctions' proteins *in vivo* and *in vitro* (Zheng et al., 2015). Additionally, we found that *TXL* could regulate gene expression in hypoxia-treated human cardiac microvascular endothelial cells (CMECs) (Li Y. N. et al., 2015) and attenuate human CMEC injury via inhibiting peroxynitrite, which contributed to macrophage-mediated human CMEC injury (Wang et al., 2015).

#### Protection against ischemia-reperfusion (I/R) injury

When AMI occurs, reperfusion of the ischemic myocardium is a key approach for limiting infarct size. However, reperfusion itself paradoxically leads to further cardiovascular complications, which is called myocardial I/R injury. Nowadays, no effective therapy is available to protect the heart from this I/R injury (Eltzschig and Eckle, 2011). Previous studies have demonstrated that *TXL* enabled to effectively protect hearts against no-reflow and reperfusion injury in a protein kinase A-dependent manner (Li et al., 2010, 2013) and ameliorate myocardial I/R injury by promoting cardiac microvascular endothelial barrier function (Qi et al., 2015). Besides, as for cerebral I/R injury, *TXL* could reduce cell death via Cx43/Calpain II/Bax/Caspase-3 pathway (Cheng et al., 2017).

#### Anti-inflammation

The immune system and inflammation, through several cellular and soluble inflammatory mediators, play a great role in the local tissue structural changes of AMI (Swirski and Nahrendorf, 2013). *TXL* was able to decrease serum contents of P-selectin, intercellular adhesion molecule 1 (ICAM-1), vascular cell adhesion molecule 1 (VCAM-1) and pro-inflammatory cytokines (interleukin 6, interleukin 10), regulate anti-inflammatory factor levels in the early reperfusion of AMI (Zhang H. T. et al., 2009). As the above result showed, *TXL* treatment group could effectively reduce the hs-CRP level and this therapeutic effect would get better along with the prolonging of treatment period. Moreover, *TXL* exerted its vascular protective effects by inhibiting the vascular inflammatory response and suppressing the gene expression of miR-155 (Zhang et al., 2014).

#### Regulation of lipid metabolism

The pathogenesis of AMI has close links with diabolism of lipids. The rise of blood fat will easily lead to the occurrence and development of vulnerable plaques, further resulting in AMI. The clinical trials indicated that *TXL* could significantly reduce the level of TC, TG, and LDL. Relevant experimental study reported that *TXL* enabled to enhance the stability of vulnerable plaques and prevent plaques from rupture in a dose-dependent manner. In addition, high-dose *TXL* group showed a low serum level of low-density lipoprotein cholesterol and ox-LDL. Besides, *TXL* was effective for decreasing serum lipid levels and inhibiting plaque inflammation in the balloon-induced abdominal aortic endothelial injury rabbits model (Chen et al., 2009).

#### Others

**Figure 9:**
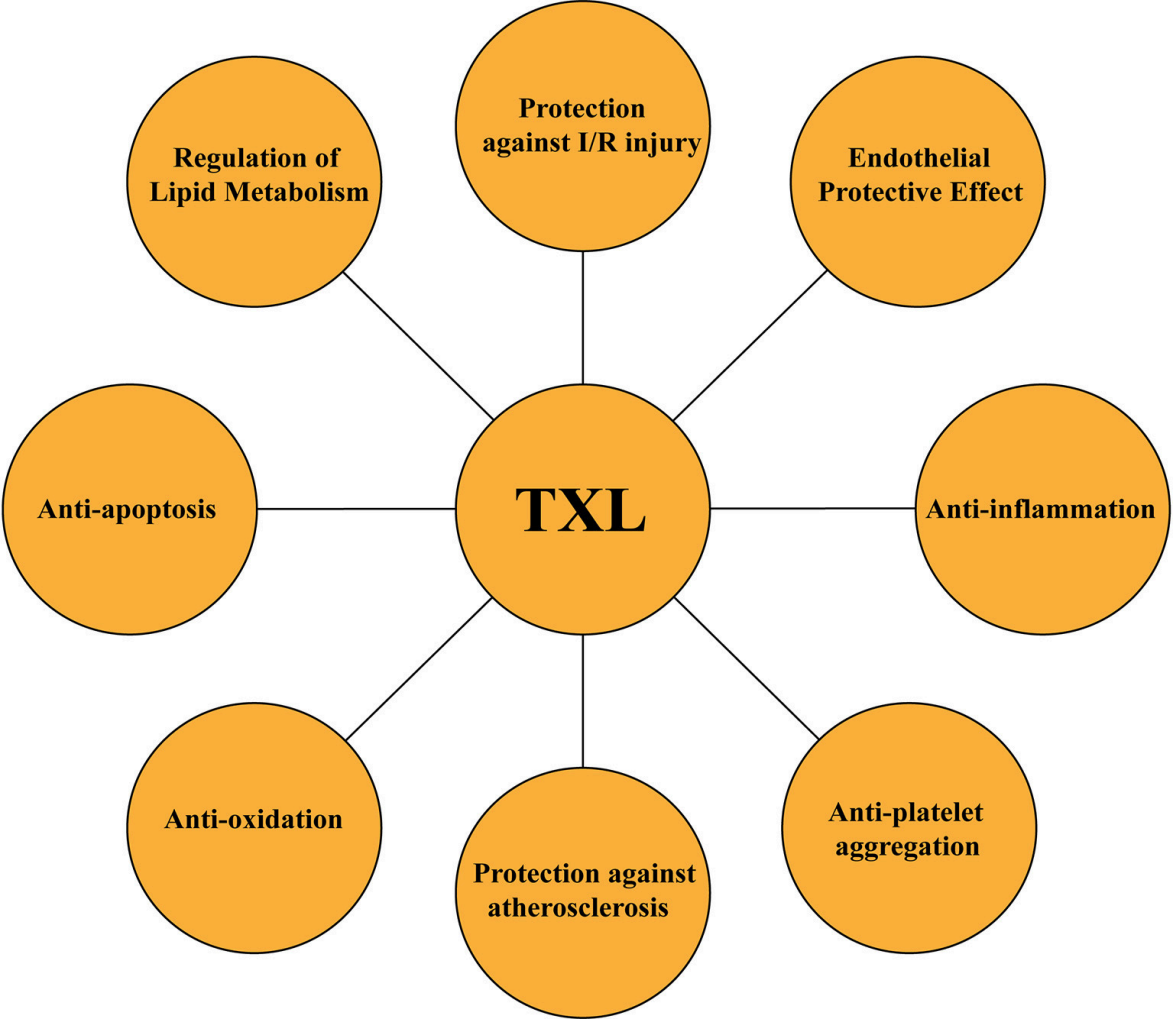
Summary of pharmacological functions of *TXL*.

A large number of studies have reported that *TXL* presented various regulatory influences on CVDs. The cardioprotective benefits of *TXL* against MI were closely related to the inhibition of apoptosis and promotion of autophagy in rat hearts after AMI by activating AMPK signaling pathway (Li Q. et al., 2017). *TXL* also exhibited anti-platelet aggregation effects (Chen et al., 2016). *TXL* may probably exhibit a specific advantage in the prevention and treatment of myocardial fibrosis via mediating the expressions of TGF-beta1, Smad3, and Smad7 in diabetic rats (Wang X. et al., 2016). Furthermore, *TXL* could inhibit OX-LDL-induced maturation of dendritic cells by regulating PPAR gamma pathway (Su et al., 2010). So, in summary, *TXL* exerted its comprehensive protective effects on myocardium and blood vessels not only in clinical trials but also in basic experiments (Figure 9).

Nevertheless, there was no statistical difference between *TXL* treatment group and control group in some fields, such as revascularization, recurrent heart failure and HDL. The reasons may include the following two aspects. Firstly, *TXL*, a compound preparation consisting of many kinds of herbs and insects, presented limited therapeutic effects on revascularization, recurrent heart failure and HDL from the current literature.

Secondly, and most importantly, the intervention time of included studies to conduct meta-analysis was less than one year, it may be difficult to observe the occurrence of revascularization and heart failure as well as the increase of HDL during the short period. Thus, long-term studies need to be further implemented.

### The safety of *TXL*

Seven studies reported adverse events in this review. Compared with the control group, the total risk rate of adverse events in the treatment group was relatively high, and the gastrointestinal discomfort accounted for the major proportion. We observed the reasons as following: ①*TXL* contains many animal medicines, such as *Leech (Hirudo, Shui Zhi), Scorpion (Scorpio, Quan Xie), Centipede (Scolopendra, Wu Gong)*, and other insect drugs, which could dredge the channel and promote blood circulation, while on the other hand, this kind of function maybe partly cause stomach irritation. ② There were a few articles reporting adverse drug reactions which was not able to comprehensively reflect the safety of *TXL*. ③ Many studies did not show adverse events of control group. Additionally, there was not statistical analysis of untoward effects in original literature, all of such reasons contributed to the large adverse events in the experimental group.

### Study limitations

With the limitation of small sample size, short-term duration and poor quality of methodology (unclear of random and blind methods), more large-scale perspective randomized double-blind long-term RCTs are needed to confirm the efficacy and safety of *TXL* in treating AMI. Furthermore, the included patients in our systematic review were all from China, so it is difficult to define the influence of race and region. Besides, there were not enough studies involving in dose-effect relationship of *TXL*.

### Clinical practice and further research of this review

Given the available evidence by our work, the results of this review suggested that *TXL* alone or combined with conventional western medicine exhibited a specific advantage in the secondary prevention of AMI, especially in the decrease of primary cardiac events. Clinical practice could consider adding oral *TXL* to routine pharmacotherapy management, it may improve the living quality of the patients and clinical efficacy. Taking *TXL* for example, it also proved that traditional Chinese medicine, a kind of complementary and alternative medicine, has its unique advantages in the treatment of some diseases. Besides, some key points need to pay more attention. Firstly, lack of detailed description of randomization and blinding was the major methodological flaw of most included studies in this review, thus, clinical experts and statistical professors should have a better cooperation to design the high-quality clinical trials and make the blinding of participants and outcome assessors. Secondly, due to the limited evidence of safety of *TXL*, more research is worth further exploring. In addition, the researchers should conduct more trials in various countries to explore the influence of different regions and races. More importantly, the biological mechanism of *TXL* and its active compounds need to further explore, which will benefit to providing more evidence for clinical treatment in CVDs.

## Conclusion

Chinese patent medicine *TXL* seemed beneficial for secondary prevention after AMI. This potential benefit needs to be further assessed through more rigorous RCTs. Furthermore, *TXL* may be safe for treating AMI due to the milder adverse events in clinic.

## Author contributions

HS and GT provided guidelines for this systematic review and meta-analysis. ML, CL, SC, and YS wrote the main manuscript and prepared the Figures 3–7, 9 and Table 2. QZ and JH conducted the literature searching, study selection and data extraction. JH provided Table 1 and Figure 1. ML and RQ assessed the risk of bias and drew Figure 2. ML and XZ assessed the GRADE and made Table 3. CZ conducted the publication bias and prepared Figure 8.

### Conflict of interest statement

The authors declare that the research was conducted in the absence of any commercial or financial relationships that could be construed as a potential conflict of interest. The reviewer JC declared a shared affiliation, though no other collaboration, with the authors to the handling Editor.
