# Supplementary material for: Potential Effectiveness of Chinese Patent Medicine Tongxinluo Capsule for Secondary Prevention After Acute Myocardial Infarction: A Systematic Review and Meta-Analysis of Randomized Controlled Trials
Source: Front Pharmacol. 2018 Aug 3;9:830. doi: 10.3389/fphar.2018.00830 (PMC6085586; doi:10.3389/fphar.2018.00830)
Supplement: Supplementary file 3 [file Table_3.DOC]

Supplementary material 3. The detail search strategy

***Pubmed. Consider the following:***

#1 Search (((((tong-xin-luo[Title/Abstract]) OR Tong-xin-luo[Title/Abstract]) OR Tong xin luo [Title/Abstract]) OR tong xin luo[Title/Abstract]) OR Tongxinluo[Title/Abstract]) OR tongxinluo [Title/Abstract]

#2 Search (((myocardial infarct*[Title/Abstract]) OR heart attack*[Title/Abstract]) OR ischemia heart disease*[Title/Abstract]) OR ischemia reperfusion[Title/Abstract]

#3 Search patient* [Title/Abstract]

#4 Search ((((((((tong-xin-luo[Title/Abstract]) OR Tong-xin-luo[Title/Abstract]) OR Tong xin luo [Title/Abstract]) OR tong xin luo[Title/Abstract]) OR Tongxinluo[Title/Abstract]) OR tongxinluo [Title/Abstract])) AND ((((myocardial infarct*[Title/Abstract]) OR heart attack*[Title/Abstract]) OR ischemia heart disease* [Title/Abstract]) OR ischemia reperfusion[Title/Abstract])) AND patient* [Title/Abstract]

***Web of Science. Consider the following:***

#1 Theme: (Tong-xin-luo) OR Theme: (tong-xin-luo) OR Theme: (Tong xin luo) OR Theme: (tong xin luo) OR Theme: (Tongxinluo) Theme: (tongxinluo)

#2 Theme: (myocardial infarct*) OR Theme: (heart attack*) OR Theme: (ischemia heart disease*) OR Theme: (ischemia reperfusion)

#3 Theme: patient*

#4 #1 AND #2 AND #3

***The Cochrane Library. Consider the following:***

#1 tong-xin-luo:ti,ab,kw or Tong-xin-luo:ti,ab,kw or Tong xin luo:ti,ab,kw or tong xin luo:ti,ab,kw or Tongxinluo:ti,ab,kw (Word variations have been searched)

#2 tongxinluo:ti,ab,kw (Word variations have been searched)

#3 (#1 or #2)

#4 myocardial infarct*:ti,ab,kw or heart attack*:ti,ab,kw or ischemia heart disease*:ti,ab,kw or ischemia reperfusion:ti,ab,kw (Word variations have been searched)

#5 patient*:ti,ab,kw (Word variations have been searched)

#6 (#3 and #4 and #5)

***China National Knowledge Infrastructure (CNKI). Consider the following:***

(AB = 'tong-xin-luo' OR AB = 'Tong-xin-luo' OR AB = 'Tong xin luo' OR AB = 'tong xin luo' OR AB = 'Tongxinluo' OR AB = 'tongxinluo') AND (AB = 'myocardial infarction' OR AB = 'heart attack' OR AB = 'ischemia heart disease' OR AB = 'ischemia reperfusion') AND (AB = 'patient*')

Noting: AB means abstract.

***WanFang Database. Consider the following:***

(abstract = 'tong-xin-luo' OR abstract = 'Tong-xin-luo' OR abstract = 'Tong xin luo' OR abstract = 'tong xin luo' OR abstract = 'Tongxinluo' OR abstract = 'tongxinluo') AND (abstract = 'myocardial infarction' OR abstract = 'heart attack ' OR abstract = 'ischemia heart disease' OR abstract = 'ischemia reperfusion') AND (abstract = 'patient*')

***Chinese Scientific Journal Database (VIP). Consider the following:***

(R = 'tong-xin-luo' OR R = 'Tong-xin-luo' OR R = 'Tong xin luo' OR R = 'tong xin luo' OR R = 'Tongxinluo' OR R = 'tongxinluo') AND (R = 'myocardial infarction' OR R = 'heart attack ' OR R = 'ischemia heart disease' OR R = 'ischemia reperfusion') AND (R = 'patient*')

Noting: R means abstract.
